# Real-World Treatment Patterns and Clinical Outcomes among Patients Receiving CDK4/6 Inhibitors for Metastatic Breast Cancer in a Canadian Setting Using AI-Extracted Data

**DOI:** 10.3390/curroncol31040161

**Published:** 2024-04-09

**Authors:** Ruth Moulson, Guillaume Feugère, Tracy S. Moreira-Lucas, Florence Dequen, Jessica Weiss, Janet Smith, Christine Brezden-Masley

**Affiliations:** 1 Pentavere, Toronto, ON M6G 1A1, Canada; 2 Pfizer Canada ULC, Kirkland, QC H9J 2M5, Canada; 3 Mount Sinai Hospital, Toronto, ON M5G 1X5, Canadachristine.brezden@sinaihealth.ca (C.B.-M.)

**Keywords:** real-world evidence, CDK4/6 inhibitors, AI, HR+, HER2−, metastatic breast cancer

## Abstract

Cyclin-dependent kinase 4/6 inhibitors (CDK4/6i) are widely used in patients with hormone receptor-positive (HR+)/human epidermal growth factor receptor 2 negative (HER2−) advanced/metastatic breast cancer (ABC/MBC) in first line (1L), but little is known about their real-world use and clinical outcomes long-term, in Canada. This study used Pentavere’s previously validated artificial intelligence (AI) to extract real-world data on the treatment patterns and outcomes of patients receiving CDK4/6i+endocrine therapy (ET) for HR+/HER2− ABC/MBC at Sinai Health in Toronto, Canada. Between 1 January 2016 and 1 July 2021, 48 patients were diagnosed with HR+/HER2− ABC/MBC and received CDK4/6i + ET. A total of 38 out of 48 patients received CDK4/6i + ET in 1L, of which 34 of the 38 (89.5%) received palbociclib + ET. In 2L, 12 of the 21 (57.1%) patients received CDK4/6i + ET, of which 58.3% received abemaciclib. In 3L, most patients received chemotherapy (10/12, 83.3%). For the patients receiving CDK4/6i in 1L, the median (95% CI) time to the next treatment was 42.3 (41.2, NA) months. The median (95% CI) time to chemotherapy was 46.5 (41.4, NA) months. The two-year overall survival (95% CI) was 97.4% (92.4, 100.0), and the median (range) follow-up was 28.7 (3.4–67.6) months. Despite the limitations inherent in real-world studies and a limited number of patients, these AI-extracted data complement previous studies, demonstrating the effectiveness of CDK4/6i + ET in the Canadian real-world 1L, with most patients receiving palbociclib as CDK4/6i in 1L.

## 1. Introduction

Breast cancer is the most common global cancer diagnosis and accounts for one out of four cancer cases and one out of six cancer deaths in females [1]. In Canada, the age-standardized mortality rate for breast cancer has declined by 48% since the 1980s due to improved screening and more effective targeted systemic therapies [2]. However, despite this trend, 5-year survival differs between stage 0–I (100%), stage II (93%), stage III (72%), and stage IV advanced/metastatic breast cancer (ABC/MBC) (22%) [3]. Following the introduction of cyclin-dependent kinase 4/6 inhibitors (CDK4/6i), palbociclib, ribociclib, and abemaciclib, over the last 8 years, CDK4/6i with endocrine therapy (ET) have become the standard of care for patients with hormone receptor-positive (HR+)/human epidermal growth factor receptor 2 negative (HER2-) ABC/MBC in first line (1L). The combination is recommended by all treatment guidelines, including the National Comprehensive Cancer Network (NCCN), the Canadian Cancer Society, and Canadian oncologists, and is supported by several phase III trials and RWE studies in the US [4,5,6,7,8,9,10,11,12,13,14]. However, there remains a lack of evidence on longer-term treatment patterns and clinical outcomes in patients with HR+/HER2− ABC/MBC in the Canadian real-world setting.

Real-world evidence (RWE) is increasingly being used to understand treatment use and outcomes in clinical practice and can complement the findings from randomized clinical trials (RCTs) [15,16,17]. For example, in the multicenter, heterogenous US cohort study by Rugo et al., (2022), palbociclib plus the aromatase inhibitor demonstrated greater median real-world progression-free survival (rwPFS) versus the aromatase inhibitor alone (19.3 [17.5–20.7] versus 13.9 [12.5–15.2] months; hazard ratio, 0.70 [95% CI, 0.62–0.78]; *p* < 0.0001), complementing PFS from the phase III PALOMA-2 study of palbociclib and letrozole versus letrozole and placebo (24.8 months [95% CI, 22.1–NA] versus 14.5 [95% CI, 12.9–17.1] months; hazard ratio, 0.58; [95% CI, 0.46 to 0.72]; *p* < 0.001) [7,12].

Recently, electronic health records (EHRs) have been leveraged as a rich source of real-world data (RWD), as they can provide a comprehensive overview of patients’ disease in a centralized location, allowing researchers to study disease progression, treatment patterns, and clinical outcomes over time. Still, complexities exist in harnessing data from the EHR. Basic patient information, such as demographics, is typically easier to collect, as it is held within structured fields of the EHR, but it may be incomplete or incorrect. Other valuable features, such as evidence of metastases, are often found within the unstructured fields, which are less easy to collect. A manual chart review is commonly used for extracting RWD from the EHR [18]. However, due to the complexities of the EHR, this is time-consuming, prone to human error, lacks scalability, and can result in inconsistent data. These challenges have contributed to the limited translation of EHR adoption into enhanced clinical care [19,20,21].

To overcome these limitations, artificial intelligence (AI) has proven its ability to extract data from structured and unstructured fields of the EHR to produce reliable, structured clinical data in a more consistent, efficient, and scalable manner compared to manual abstraction [18,22,23]. This technology allows clinicians and researchers to access previously unavailable RWD and is being used for patient and disease identification, pharmacovigilance, and the development of learning health systems [23,24,25,26,27,28].

Complexities also exist for the AI extraction of RWD from the EHR as a result of inconsistencies in the sections of the EHR where information is stored, variations and complexity in the narrative used within clinical text, and the need to coordinate multiple pieces of evidence temporally. This can result in uncertainty regarding the validity and transferability of such technologies [29]. The commercially available AI engine, DARWEN^TM^ (Darwen, UK), has been evaluated against manual abstraction for the same clinical features in multiple disease areas, including breast cancer [25], lung cancer [18,30,31,32,33,34], ambulatory care diseases [23], and dermatology [28] at multiple Canadian institutions, validating its use to extract RWD more accurately and efficiently than a manual chart review. Sinai Health is a leading Canadian cancer center and has been using EHR systems since 2006 with the goal of leveraging technology to harness data from the EHRs to inform clinician decision-making.

In this study, we describe how the AI extraction of RWD was used to describe and better understand the treatment patterns and clinical outcomes of Canadian patients receiving CDK4/6i + ET for HR+/HER2− ABC/MBC in a real-world setting, with a longer follow-up. RWE is necessary to understand these trends to inform targeted sequencing and future treatment decisions in this population. 

## 2. Materials and Methods

### 2.1. Study Design

This was a retrospective chart review of the data from the EHRs of patients diagnosed with HR+/HER2− ABC/MBC between 1 January 2016 and 1 July 2021, receiving CDK4/6i treatment at Sinai Health, Toronto. Included patients were as follows: women aged ≥ 18 years old, diagnosed with HR+/HER2− ABC/MBC between 1 January 2016 and 1 July 2021, and treated with CDK4/6i. The study period encompassed 1 January 2016 to 1 October 2021 to capture all patients treated with CDK4/6i since their approval and allowed for a minimum three-month follow-up period.

### 2.2. Clinical Feature Extraction

The clinical features extracted from Sinai Health’s EHRs included patient demographics, clinical characteristics, treatment information, and clinical outcomes. Data were extracted from the patient EHRs using DARWEN^TM^ AI technology, or for three specific features (radiation treatment, date of ABC/MBC diagnosis, and treatment start/stop date), the data were extracted manually. Finally, some features were derived using the extracted data, such as age at ABC/MBC diagnosis and clinical outcomes, including the time to the next treatment (TTNT), time to chemotherapy (TTC), and overall survival (OS).

DARWEN^TM^—which has been previously described and validated in detail—combines multiple state-of-the-art approaches to extract relevant data from structured and unstructured EHR fields [18,23]. DARWEN^TM^ uses a “twin-engine design”, which allows model training to begin on one task while learnings and adjustments can be made quickly and easily for adjacent tasks. This provides knowledge transfer between tasks, flexibility, and adaptability, reducing the overall number of models required and hence the compound error, thus achieving high accuracy with the results that are aligned with clinician expertise.

All features were extracted following pre-defined rules and definitions developed by the Sinai Health Principal Investigator (PI). Based on the reality of the available data at Sinai Health, the definitions and rules were updated in an iterative process until a finalized set of rules was agreed upon with the PI. A full list of the features extracted, as well as the feature definitions and data sources, can be found in Supplementary Table S1.

For the features extracted from the unstructured EHR field, DARWEN^TM^ algorithms were pre-trained on general medical and other ABC/MBC datasets and then fine-tuned and validated on the Sinai Health data, as detailed below.

**Figure 1:**
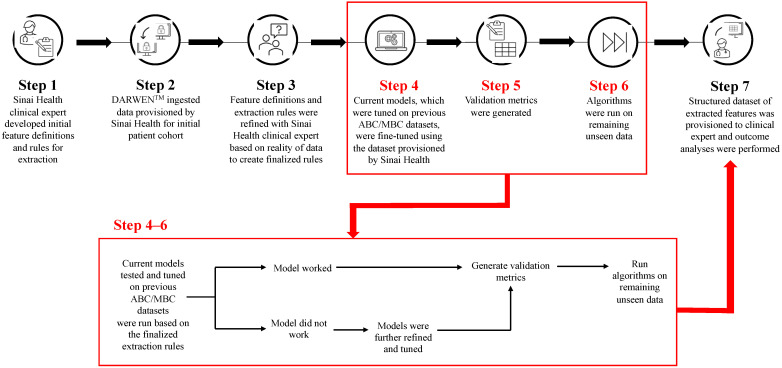
Workflow and methodology used to refine, test, and validate models. ABC/MBC: Advanced/metastatic breast cancer.

Using the initial data provisioned by Sinai Health (which included all patients at Sinai Health who received a CDK4/6i and were aged ≥ 18 years), one subset of patient data was used for the fine-tuning and testing of the algorithms based on the finalized feature definitions and extraction rules, until accuracy, precision (positive predictive value), recall (sensitivity), and F1 (the harmonic mean of precision and recall) score targets were achieved. The AI training and tuning methods have previously been reported [18]. Subsequently, the models were applied to a second subset of data (distinct from the first one) to generate validation metrics against data unseen by the model. The steps were repeated if necessary until the results on both subsets met the target scores and were sufficiently stable. Finally, the models were run on all the remaining data, which had not been part of either the first or second subset, to produce the final dataset. See Figure 1 for the workflow and methodology used throughout this study. All extracted data (irrespective of the extraction method) was reviewed by the PI to confirm that the findings aligned with their clinical expectations.

### 2.3. Outcomes

The primary outcome was to characterize real-world treatment patterns among patients with HR+/HER2− ABC/MBC receiving CDK4/6i. Other outcomes of interest included clinical outcomes: TTNT for 1L, TTC from diagnosis, and OS. TTNT was measured from the date of the initiation (first dose) of treatment to the date of the initiation of the subsequent line of therapy. Patients who did not progress on to a subsequent line of therapy were censored at their last known date of treatment. TTC was measured from the date of ABC/MBC diagnosis to the date of chemotherapy initiation. Patients still on treatment and who did not start chemotherapy were censored at the date of their last follow-up or death, whichever came first. Patients who died before starting their next line of therapy were also censored. OS was measured from the date of ABC/MBC diagnosis to the date of death. For patients where no death event was found, the date of the last follow-up was used, and these patients were censored in the survival analyses.

### 2.4. Statistical Analyses

Descriptive analyses summarized patients’ demographics, clinical characteristics, and outcomes of interest across the study cohort. Continuous variables were described using mean and standard deviation (SD), median, and the first and third quartiles. Categorical variables were described by frequencies and percentages. Kaplan–Meier (KM) curves were used to describe the time to event(s) and followed standard censoring rules.

## 3. Results

DARWEN^TM^ was used to extract nine features found within the unstructured fields of the EHR. AI performance for the extracted features is shown in Supplementary Table S2. An F1 score (the harmonic mean of precision and recall) of 1.00 was achieved for three features: histology, ER receptor status, and PR receptor status, and an overall accuracy (the number of correctly identified predictions) of above 90% was achieved for all AI-extracted features. These results are consistent with the previous validations of DARWEN^TM^ [18,23]. Radiation treatment, the date of ABC/MBC diagnosis, and treatment (start/stop date) were extracted manually due to the limitations imposed by the data captured in the EHR. Radiation treatment is administered at sites outside of Sinai Health; therefore, information on a patient’s radiation therapy was not consistently captured in the Sinai Health patient EHR. The date of the ABC/MBC diagnosis is also often inconsistently reported in the patient’s EHR, with the ABC/MBC diagnosis often being reported as suspicious but not confirmed. Additionally, patients were often diagnosed with ABC/MBC at other sites and referred to Sinai Health. Prescription information is not stored electronically in the EHR system at Sinai Health but rather in paper format, dictated into clinical notes. Before data extraction using either method, the pre-defined rules and definitions for each clinical feature were finalized with the Sinai Health PI (Supplementary Table S1).

### 3.1. Patients

In total, DARWEN^TM^ ingested a total of 5052 patient reports, including clinical, pathology, and radiology reports for 87 patients at Sinai Health who received a CDK4/6i and were aged ≥ 18 years. A total of 48 patients were identified as having HR+/HER2− ABC/MBC diagnosed between 1 January 2016 and 1 July 2021 and were treated with a CDK4/6i during the study period.

The baseline characteristics for the 48 included patients can be found in Table 1. In this cohort, the median age was 60.5 years. The majority of patients (70.8%) had recurrent ABC/MBC, and 29.2% had de novo disease; 66.7% of the patients had ductal carcinoma, and 18.8% were pre-menopausal. A total of 31.2% of patients presented with bone-only metastases at their ABC/MBC diagnosis. A total of 39.6% of patients had lung metastases during the study period, and 37.5% had liver metastases during the study period. A total of 45.8% of patients had one metastatic site during the study period. Of the patients with reported Eastern Cooperative Oncology Group (ECOG) performance scores at diagnosis (22/48), the majority had an ECOG score of 0/1 (18/22 [81.8%]). At ABC/MBC diagnosis, the most common comorbidity was hypertension (37.5%), followed by diabetes (14.6%). The tumor grade at ABC/MBC diagnosis was not consistently reported across the patients, with 29 out of 48 (60.4%) missing tumor grades at the time of their ABC/MBC diagnosis. Of the 48 patients, 38 received a CDK4/6i in the 1L setting. Baseline demographics for the 38 patients who received a CDK4/6i in 1L were similar to the full patient cohort (Table 1). Of the full cohort, 21 out of 48 (43.8%) patients went on to receive a second line (2L) therapy during the study, and 12 of the 48 (25.0%) went on to receive a third line (3L) therapy during the study.

### 3.2. Treatment Patterns

Treatment patterns were assessed from the date of ABC/MBC until the date of death, date of last follow-up, or the end of the study period, whichever came first (the median duration of follow-up for all patients was 28.7 months). Throughout the study period, across all patients, CDK4/6i included abemaciclib, palbociclib, and ribociclib. ET included tamoxifen, anastrozole, letrozole, exemestane, and fulvestrant. Chemotherapy included the following agents (either a single agent or in combination): capecitabine, cisplatin, cyclophosphamide, paclitaxel, docetaxel, doxorubicin, eribulin, and/or gemcitabine.

Of 38 out of 48 patients who received a CDK4/6i in 1L, 34 of 38 (89.5%) received palbociclib + ET (Table 2; Supplementary Figure S1). Letrozole was the most common ET given with CDK4/6i in 1L (30/38 [78.9%]). A total of 27 out of 48 (56%) patients did not go on to receive a 2L during the study period (for reasons including that the patient remained on 1L, the patient died, or the patient was lost to follow-up). Of the 21 patients who went on to 2L treatment during the study period, 12 out of 21 (58.3%) of these patients received a CDK4/6i, of which 7 out of 12 received abemaciclib + ET (Table 2; Supplementary Figure S1). Fulvestrant was the most commonly prescribed ET with CDK4/6i in 2L (9/12 [75.0%]). The majority of patients who progressed to a 3L therapy received chemotherapy (10/12 [83.3%]) (Table 2; Supplementary Figure S1).

### 3.3. Clinical Outcomes

**Figure 2:**
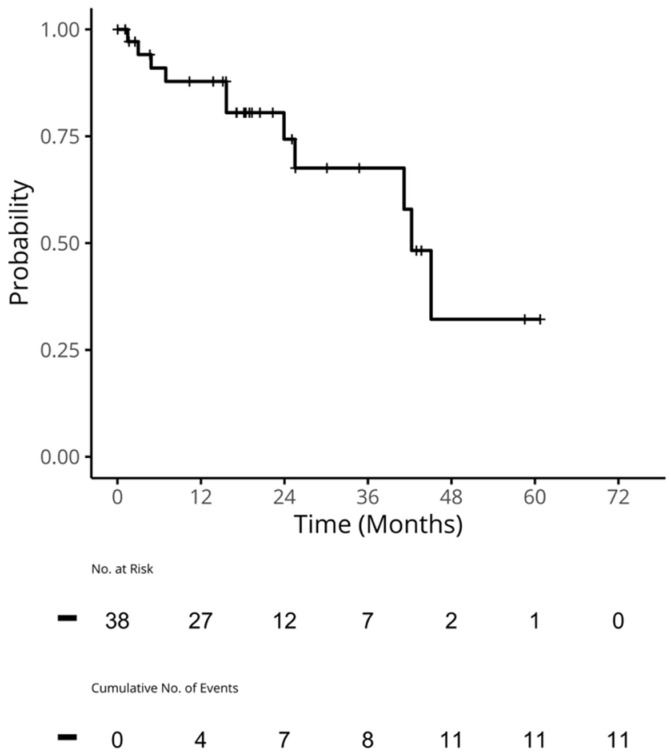
TTNT1 in patients who received a CDK4/6i in 1L. Median TTNT (95% CI): 42.3 (41.2, NA) months. CDK4/6i: cyclin-dependent kinase 4/6 inhibitors; CI: confidence interval; NA: not applicable; TTNT1: the time to next treatment was calculated by subtracting the start date of 1L from the start date of 2L. Patients who did not go on to receive 2L were censored at their last known date of treatment.

**Figure 3:**
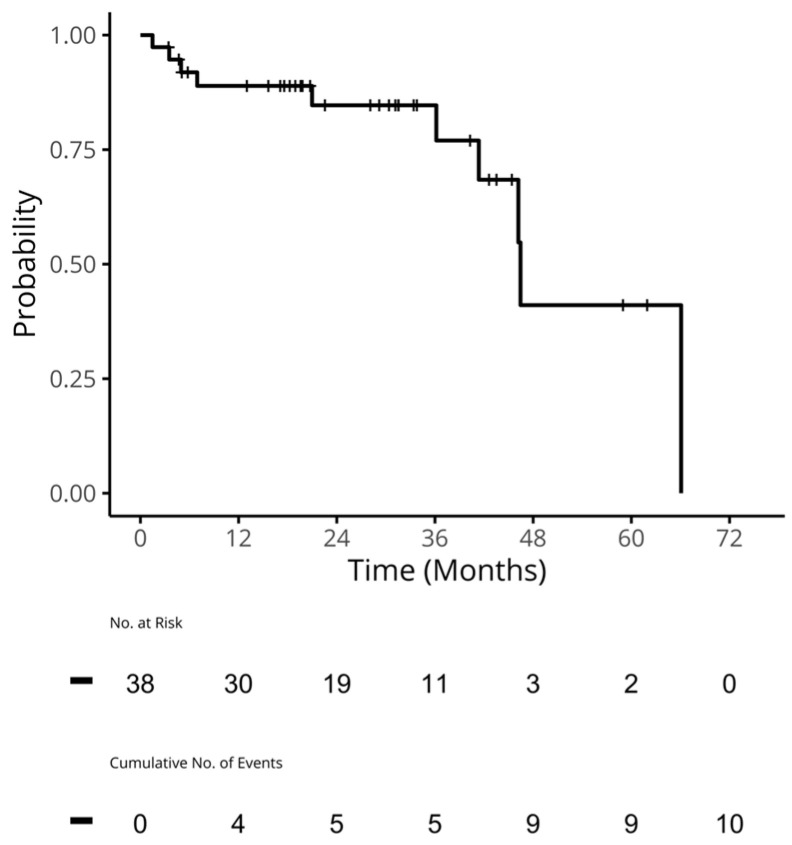
TTC in patients who received a CDK4/6i in 1L. Median (95% CI) TTC: 46.5 (41.4, NA) months. CDK4/6i: cyclin-dependent kinase 4/6 inhibitors; CI: confidence interval; NA: not applicable; TTC: the time to chemotherapy was calculated by subtracting the start date of chemotherapy from the date of ABC/MBC diagnosis. Patients who did not receive chemotherapy were censored at their date of last follow-up or death. Patients who experienced death before starting their next treatment were also censored.

**Figure 4:**
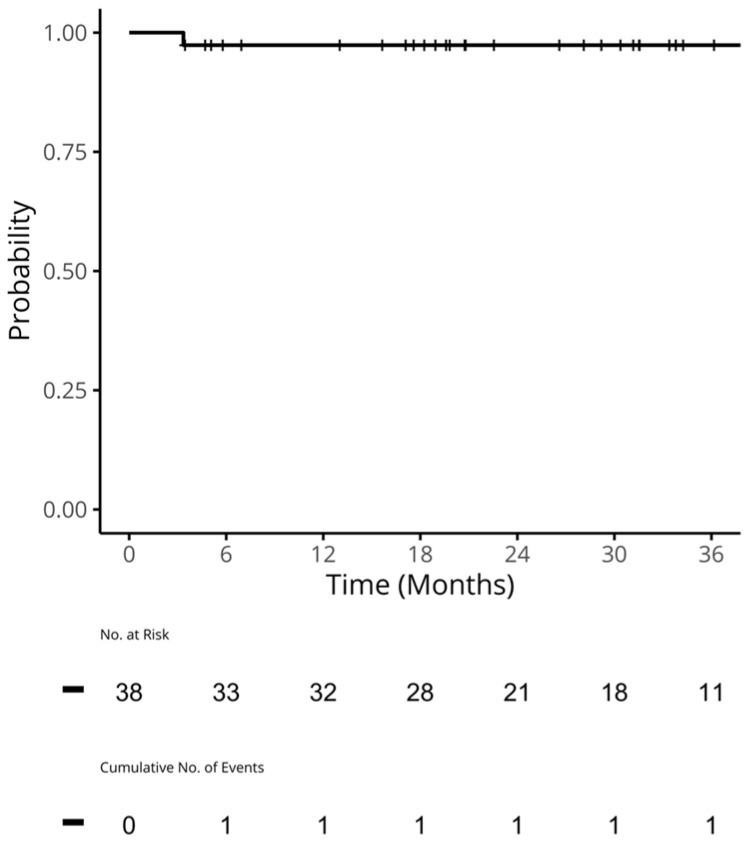
OS in patients who received a CDK4/6i in 1L. Median OS (95% CI): NA (NA, NA) months. Two-year OS (95% CI) was 97.4% (92.4, 100.0). CDK4/6i: cyclin-dependent kinase 4/6 inhibitors; CI: confidence interval; NA: not applicable; OS: the overall survival was calculated by subtracting the date of starting 1L from the date of death. Patients who did not die were censored at their last date of follow-up or the study’s end date.

For the patients who received a CDK4/6i in 1L, the median (95% confidence interval [CI]) time to the next treatment for 1L (TTNT1) was 42.3 (41.2, NA) months (Figure 2). The median (95% CI) TTC for these patients was 45.1 months (41.2, NA; Figure 3). A median (95%) OS was not reached, and the 2-year OS rate (95% CI) was 97% (92%, 100%; Figure 4).

## 4. Discussion

This study illustrates the validity of using AI technologies for identifying patients with HR+/HER2− ABC/MBC and generating RWE, including the treatment patterns and clinical outcomes for patients. AI was used to extract nine crucial features from the patient EHR, which were validated and reviewed by a breast cancer expert. The results from this study complement the findings from previous RWE studies and demonstrate the effectiveness of CDK4/6i in the Canadian real-world 1L setting (particularly palbociclib, as most patients in this study received 1L palbociclib) over a longer follow-up period than previous real-world Canadian studies (up to 69 months versus 62 and 24) [35,36].

Recently, much progress has been made in the implementation of AI tools in healthcare, including assisting radiologists in detecting abnormalities and disease from X-rays, MRIs, and CT scans, personalized medicine and predicting which treatments are likely to benefit a patient, clinical decision support systems and AI-remote monitoring and telemedicine platforms [37,38,39]. Additionally, AI tools used for the extraction of clinical text can make sense of and analyze vast amounts of unstructured clinical text from pathology reports, clinical notes, and radiology reports. These tools, such as DARWEN^TM^, are being used for patient and disease identification, pharmacovigilance, and the development of learning health systems [23,24,25,26,27,28]. However, many tools, such as ClinicalBERT, rely on open-source datasets, such as the MIMIC-III dataset of de-identified hospital records from intensive care units [40,41]. These datasets have limited insight into the entirety of the patient journey and may not be appropriate for investigating diseases such as breast cancer, for which care is provided in many different settings outside of the intensive care unit and over long periods of time. Further, many of these tools only focus on a single clinical feature, e.g., a diagnosis of a certain condition or the development of metastases, with few investigating multiple distinct medical features [42,43,44]. In comparison, this study investigated multiple complex features throughout the patient’s journey, which are critical for determining knowledge gaps and unmet needs for patients with breast cancer.

While AI holds immense promise in improving cancer diagnosis, treatment, and outcomes, it is important to recognize the challenges and limitations of the technology, specifically related to accuracy and precision. AI algorithms are only as reliable as the data they are trained on, and biases in training data can lead to inaccurate outcomes, particularly in underrepresented populations. In the context of this study, limitations imposed by the data captured in the EHR were observed, which hindered the ability to extract certain features using a completely AI approach. For example, the administration of radiation treatment outside of Sinai Health resulted in inconsistent reporting of such treatment within the clinical notes. This inconsistency posed challenges for AI in capturing all instances of when the patients received radiation therapy. However, the incomplete documentation presented similar difficulties for manual abstractors. Additionally, prescription information is not stored electronically in the EHR system at Sinai Health, but rather in paper format, dictated into clinical notes. Consequently, these records are susceptible to missingness, incompleteness, and human error. Notably, it was found that clinicians tended to document the initiation of treatment more consistently than its termination. Limited use of imputation methods was used for missing treatment dates; however, if only the month and year were present for a date, then “15” was input as the date to create a complete observation. The date of death was also not consistently reported in the EHR, as these data are only collected when hospitals are notified of a patient’s death and are provided with the exact date. These limitations are consistent with previous applications of AI tools for the extraction of oncology EHR data, but it is important to note that these limitations also impact the manual curation of data, highlighting a broader limitation in generating RWE from EHR systems [18,45].

Currently, at Sinai Health and other Canadian institutions, more sophisticated and universal EHR systems are being implemented (e.g., EPIC and Cerner Solutions), which will likely improve the ability of AI to extract data efficiently and accurately for generating RWE. AI data extraction from EHRs could allow institutions such as Sinai Health to more quickly and easily understand how they are performing compared to the currently published metrics, enabling them to perform meaningful QA projects and enhance patient care.

As this study was conducted at a single institution, there was a limited number of eligible patients, and in accordance with the hospital data privacy regulations in place at Sinai Health, observations that included less than or equal to five patients were suppressed. Future studies may hope to include additional sites to increase the number of patients included and potentially increase the diversity of patient cases represented in the results. Future work at Sinai Health may also hope to expand the use of AI technologies to harness data from the EHR system across breast cancer cohorts and disease domains and for further applications, such as patient and disease identification and the development of learning health systems, for ongoing prospective data collection.

Despite the limitations inherent to RWE studies using EHR data and the limited sample size, the real-world clinical outcomes observed in this study complemented those previously reported in the US and Canada. For Canadian patients receiving a C DK4/6i in 1L (97.8% of whom received palbociclib + ET), the 1-year OS was 97% (92%, 100%). This is similar to the 1-year survival rate reported in the Ibrance^®^ Real World Insights Study (IRIS) (the 1-year survival rate was 95.6% for palbociclib + AI and 100% for palbociclib and fulvestrant) [35]. Further, the median (95% CI) for TTNT1 was 42.3 (41.2, NA) months for patients receiving a CDK4/6i in 1L, which is longer than the median rwPFS for palbociclib combination treatment from the US DeMichele et al. (2022) study (20.0 months [95% CI, 17.5–21.9]) for 1L [11]. Additionally, the validation metrics for AI-extracted data are consistent with the previous validations of DARWEN^TM^, which has been evaluated against a manual abstraction for the same clinical features in breast cancer [25], lung cancer [18,30,31,32,33,34], ambulatory care diseases [23], and dermatology [28] at multiple Canadian institutions.

## 5. Conclusions

This study highlights the validity of AI technology in identifying patients with HR+/HER2− ABC/MBC and generating RWE, including treatment patterns and clinical outcomes for patients. This type of technology allows for a more efficient, consistent, and scalable extraction of data from EHR systems. AI was used to extract nine crucial features from the patient EHR, which were validated and reviewed by a breast cancer expert, and the accuracy metrics were consistent with the previous validations of the AI technology. The results from this study demonstrate the effectiveness of CDK4/6i + ET in the Canadian real-world 1L, with most patients receiving palbociclib as the CDK4/6i in 1L over a longer follow-up period than in previous real-world Canadian studies.

## Figures and Tables

**Table 1:** Demographics and baseline characteristics of all patients and patients who received a CDK4/6i in 1L.

	All Patients (N = 48)	Patients Receiving CDK4/6i in 1L (N = 38)
**Age at ABC/MBC diagnosis**		
Mean (SD)	57.9 (14.0)	58.4 (13.0)
Median	60.5	61.0
Q1, Q3	48.8, 67.0	50.0, 65.5
Range	23.0–89.0	23.0–84.0
**Year of ABC/MBC diagnosis ^a^**		
2016–2018	20 (41.7%)	16 (42.1%)
2019–2021	28 (58.3%)	22 (57.9%)
**Sex**		
Female	48 (100.0%)	38 (100.0%)
**Tumor histology**		
Ductal	32 (66.7%)	25 (65.8%)
Lobular	7 (14.6%)	≤5 (NR)
Mixed	≤5 (NR)	≤5 (NR)
Other	8 (16.7%)	8 (21.1%)
**De novo/recurrent at initial BC diagnosis**		
De novo	14 (29.2%)	10 (26.3%)
Recurrent	34 (70.8%)	28 (73.7%)
**HER2 status at ABC/MBC diagnosis**		
Negative	48 (100.0%)	38 (100.0%)
**ER status at ABC/MBC diagnosis**		
Positive	48 (100.0%)	38 (100.0%)
**PR status at ABC/MBC diagnosis**		
Negative	13 (27.1%)	11 (28.9%)
Positive	30 (62.5%)	23 (60.5%)
Unknown	≤5 (NR)	≤5 (NR)
**ECOG at ABC/MBC diagnosis**		
0	6 (12.5%)	≤5 (NR)
1	12 (25.0%)	10 (26.3%)
2	≤5 (NR)	≤5 (NR)
3	≤5 (NR)	≤5 (NR)
Unknown	26 (54.2%)	21 (55.3%)
**Tumor grade at ABC/MBC diagnosis**		
1	≤5 (NR)	≤5 (NR)
2	11 (22.9%)	8 (21.1%)
3	≤5 (NR)	≤5 (NR)
Unknown	29 (60.4%)	24 (63.2%)
**Organ-level metastatic sites ^b^**		
Bone	35 (72.9%)	26 (68.4%)
Bone-only metastases	15 (31.2%)	12 (31.6%)
Brain	≤5 (NR)	≤5 (NR)
Lung	19 (39.6%)	16 (42.1%)
Liver	18 (37.5%)	12 (31.6%)
**Number of metastatic sites during study period**		
0	≤5 (NR)	≤5 (NR)
1	22 (45.8%)	19 (50.0%)
2	14 (29.2%)	9 (23.7%)
3	≤5 (NR)	≤5 (NR)
4	≤5 (NR)	≤5 (NR)
**Comorbidities at ABC/MBC diagnosis ^b^**		
Atrial Fibrillation	≤5 (NR)	≤5 (NR)
Hypertension	18 (37.5%)	12 (31.6%)
Diabetes	7 (14.6%)	≤5 (NR)
Coronary Artery Disease	≤5 (NR)	≤5 (NR)
**Radiotherapy for ABC/MBC ^b^**		
Any radiotherapy	18 (37.5%)	13 (34.2%)
**Follow-up since diagnosis (months)**		
Mean (SD)	28.8 (16.7)	28.7 (16.9)
Median	29.3	28.7
Q1, Q3	17.5, 37.2	17.8, 39.3
Range	3.4–67.6	3.4–67.6

^a^ 2016–2018 represents the first half of the study period, and 2019–2021 represents the second half of the study period. ^b^ Denominator for the table is the patient population number. Percentages will not add up to 100%, as some patients may have multiple values. Pre-menopausal was defined as patients who are 50 years old or younger and are on an LHRH antagonist at any point. ABC/MBC: advanced/metastatic breast cancer; CDK4/6i: cyclin-dependent kinase 4 and 6 inhibitors; ECOG: Eastern Cooperative Oncology Group; LHRH: luteinizing hormone-releasing hormone; NR: not reported (data are suppressed to protect privacy, as per site’s requirement); SD: standard deviation.

**Table 2:** Treatment patterns for all patients across 1L, 2L, and 3L of treatment.

1L Treatment Regimen	All Patients (N = 48)
Palbociclib + ET	30 (62.5%)
Palbociclib + ET + goserelin	≤5 (NR)
Other CDK4/6i + ET	≤5 (NR)
Other CDK4/6i + ET + goserelin	≤5 (NR)
ET	≤5 (NR)
Chemotherapy	7 (14.6%)
**2L treatment regimen**	**All patients (N = 21)**
Palbociclib + ET	≤5 (NR)
Palbociclib + ET + goserelin	≤5 (NR)
Other CDK4/6i + ET	7 (33.3%)
Other CDK4/6i + ET + goserelin	≤5 (NR)
Alpelisib + ET	≤5 (NR)
ET	≤5 (NR)
Chemotherapy	6 (28.6%)
**3L treatment regimen**	**All patients (N = 12)**
Palbociclib + ET + goserelin	≤5 (NR)
Other CDK4/6i	≤5 (NR)
Chemotherapy	10 (83.3%)

CDK4/6i: cyclin-dependent kinase 4 and 6 inhibitors; ET: endocrine therapy; NR: not reported (data are suppressed to protect privacy, as per site’s requirement).

## Data Availability

The data presented in this study are not publicly available due to the privacy of individuals. The data presented in this study may be available upon reasonable request from the senior author.

## Supplementary Materials

The following supporting information can be downloaded at: https://www.mdpi.com/article/10.3390/curroncol31040161/s1, Table S1: Clinical features extracted, definitions, and data sources; Table S2: Evaluation of AI algorithms compared with manual chart review; Figure S1: Sankey diagram of treatment patterns in all patients.
