# Supplementary material for: Real-World Treatment Patterns and Clinical Outcomes among Patients Receiving CDK4/6 Inhibitors for Metastatic Breast Cancer in a Canadian Setting Using AI-Extracted Data
Source: Curr Oncol. 2024 Apr 9;31(4):2172–84. doi: 10.3390/curroncol31040161 (PMC11049664; doi:10.3390/curroncol31040161)
Supplement: Supplementary file 1 [file curroncol-31-00161-s001.zip › curroncol-2904178-supplementary.pdf]

Supplementary Materials

Real-World Treatment Patterns and Clinical Outcomes Among Patients Receiving CDK4/6 Inhibitors for Metastatic Breast Cancer in a Canadian Setting Using AI-Extracted Data

Breast Cancer Research and Treatment

Table S1. Clinical features extracted, definitions and data sources

Feature (possible output)	Feature definition	Data source
Date of birth (YYYY-MM-DD)	Patient's date of birth as documented in structured format	Structured EHR field
Sex (Male, Female)	Patient's sex as documented in structured format	Structured EHR field
Date of death (YYYY-MM-DD)	Patient's date of death as documented in structured format	Structured EHR field
Date of last follow-up (YYYY-MM-DD)	Patient's date of last follow-up as documented in structured format	Structured EHR field

Feature (possible output)	Feature definition	Data source
Date of ABC/MBC diagnosis (YYYY-MM-DD)	First mention in patient records of positive diagnosis of advanced or metastatic breast cancer	Unstructured patient clinical notes, pathology reports or radiology reports
ECOG at ABC/MBC diagnosis (0–5)	ECOG score reported closest to date of ABC/MBC diagnosis (up to 5 months pre/post-ABC/MBC diagnosis)	Unstructured patient clinical notes
Tumour grade at ABC/MBC diagnosis (1–3)	Highest reported tumour grade closest to date of ABC/MBC diagnosis (up to 5 months pre/post-ABC/MBC diagnosis)	Unstructured patient clinical notes and pathology reports
Histology (Lobular, Ductal, DCIS, LCIS, Mixed, Other)	Breast cancer histology reported closest to date of ABC/MBC	Unstructured patient clinical notes and pathology reports
De novo/recurrent status (De novo, Recurrent)	De novo: Metastases discovered in first 3 months of initial breast cancer diagnosis; Recurrent: Metastases discovered more than 3 months from initial breast cancer diagnosis	Unstructured patient clinical notes
Metastatic sites (Bone, Brain, Lung, Liver)	First positive mention of metastases from conclusions, summary, impression, interpretation or findings section of radiology report or clinical notes	Unstructured patient clinical notes and radiology reports

Feature (possible output)	Feature definition	Data source
HER2 status (Positive, Negative, Unknown, Indeterminate)	HER2 status reported closest to date of ABC/MBC	Unstructured patient clinical notes and pathology reports
ER status (Positive, Negative, Unknown, Indeterminate)	ER status reported closest to date of ABC/MBC	Unstructured patient clinical notes and pathology reports
PR status (Positive, Negative, Unknown, Indeterminate)	PR status reported closest to date of ABC/MBC	Unstructured patient clinical notes and pathology reports
Comorbidities (Atrial Fibrillation, Hypertension, Coronary Artery Disease, Diabetes, Stroke)	Positive diagnosis of comorbidities of interest	Unstructured patient clinical notes
Radiation treatment for ABC/MBC (Yes, No)	Any positive mention of radiation therapy of interest[a] after the date of ABC/MBC diagnosis	Unstructured patient clinical notes
Systemic therapy and concomitant LHRH agonists (drug name, start date, end date, line of therapy)	Any systemic therapy or concomitant LHRH agonist administered after date of ABC/MBC and associated start and stop date. Lines of therapy were based on treatment start and stop dates, combining therapies into a single line when dates overlap	Unstructured/derived from patient clinical notes
Premenopausal status (Yes, No)	Premenopausal was defined as patients who were 50 years or younger and were on an LHRH agonist at any point.	Derived

Feature (possible output)	Feature definition	Data source
Age at ABC/MBC diagnosis	Patient's age at ABC/MBC diagnosis; calculated using patient's date of birth and date of ABC/MBC diagnosis	Derived
TTNT1	Duration of 1L of therapy; calculating using systemic therapy start and stop dates[b]	Derived
TTC	Time from date of ABC/MBC to initiation chemotherapy; calculated using date of ABC/MBC diagnosis and start date of chemotherapy[c]	Derived
OS	Overall survival; calculated using date of ABC/MBC diagnosis and date of death[d]	Derived

[a]Included WBRT, SRS, SBRT, PROP or Other; [b]TTNT was calculated by subtracting start date of 1L from the start date of 2L. Patients who did not go on to receive 2L were censored at their last known date of treatment; [c]TTC was calculated by subtracting start date of chemotherapy from the date of ABC/MBC diagnosis. Patients who didn't receive chemotherapy were censored at their date of last follow-up or death. Patients who experience death before starting their next treatment were also censored; [d]OS was calculated by subtracting the date of starting 1L from the date of death. Patients who did not die were censored at their last date of follow-up or study end date. ABC/MBC: Advanced/metastatic breast cancer; DCIS: Ductal carcinoma in situ; ECOG: Eastern Cooperative Oncology Group; ER: Estrogen receptor; HER2: Human Epidermal Receptor Growth Factor 2; LCIS: Lobular carcinoma in situ; LHRH: Luteinizing hormone-releasing hormone; OS: Overall survival; PR: Progesterone Receptor; PROP: Palliative radiation oncology program; SBRT: Stereotactic body radiation therapy; SRS: Stereotactic radiosurgery; TTC: Time from date of ABC/MBC to starting chemotherapy; TTNT1: Duration of treatment for 1L; WBRT: Whole brain radiation therapy.

Table S2. Evaluation of AI algorithms compared with manual chart review

Feature	Possible values	Precision	Recall	F1	Overall accuracy
Histology	Lobular, Ductal, DCIS, LCIS, Mixed, Other	1.00	1.00	1.00	1.00

Feature	Possible values	Precision	Recall	F1	Overall accuracy
ECOG at ABC/MBC diagnosis	0–5	0.96	0.96	0.96	0.96
Metastatic sites	Brain, Bone, Liver, Lung	0.97	0.98	0.97	0.97
Tumour grade at ABC/MBC diagnosis	1–3	0.94	0.94	0.94	0.94
Comorbidities	Atrial Fibrillation, Hypertension, Coronary Artery Disease, Diabetes, Stroke	0.94	0.94	0.94	0.94
HER2 receptor status	Positive, Negative, Indeterminate, Unknown	0.88	0.88	0.88	0.94
ER receptor status	Positive, Negative, Indeterminate, Unknown	1.00	1.00	1.00	1.00
PR receptor status	Positive, Negative, Indeterminate, Unknown	1.00	1.00	1.00	1.00
De novo/recurrent status	De novo, recurrent	0.95	0.95	0.95	0.95

ABC/MBC: Advanced/metastatic breast cancer; DCIS: Ductal carcinoma in situ; ECOG: Eastern Cooperative Oncology Group; ER: Estrogen receptor; HER2: Human Epidermal Receptor Growth Factor 2; LCIS: Lobular carcinoma in situ; PR: Progesterone Receptor.

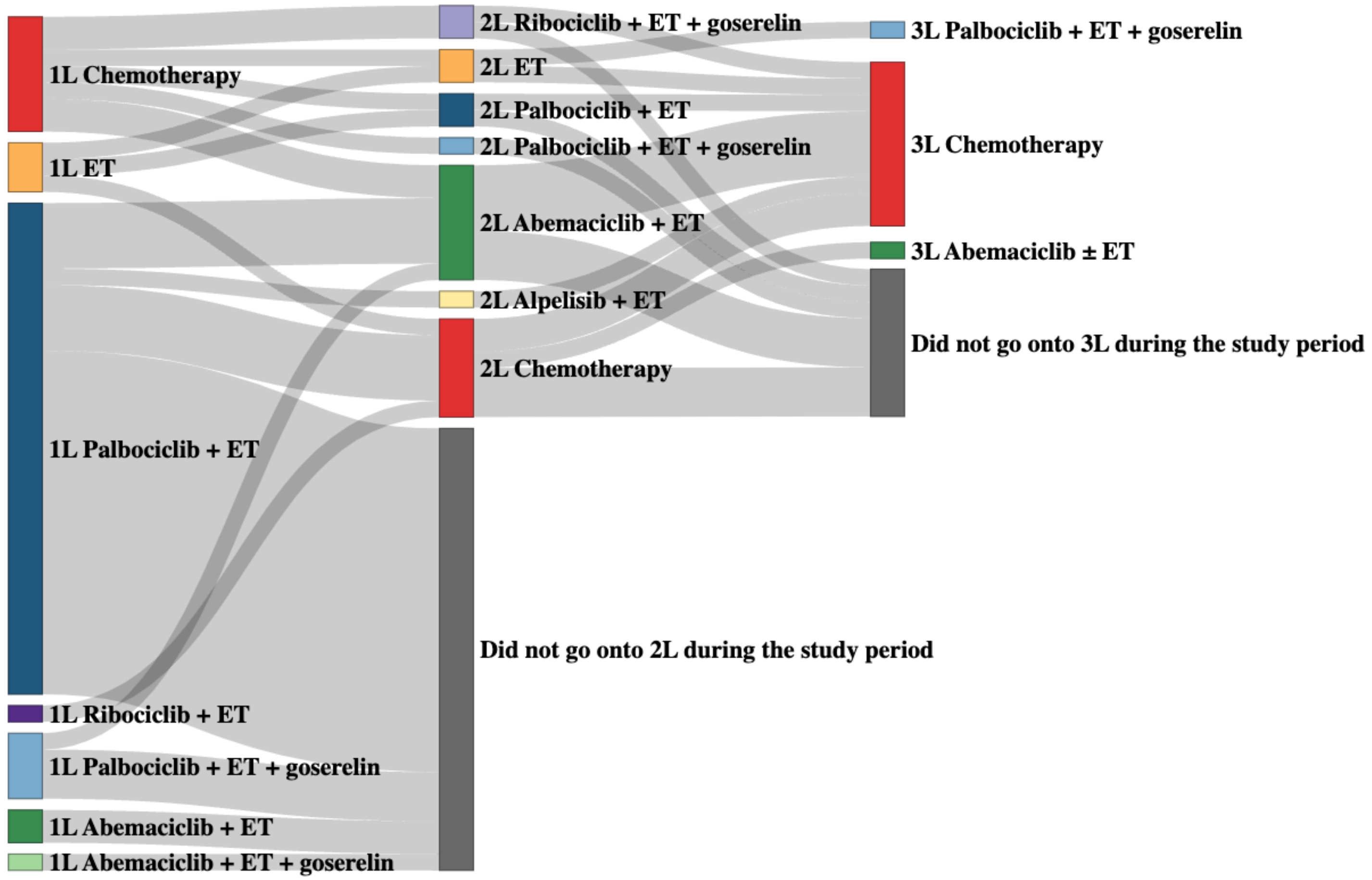

Figure S1. Sankey diagram of treatment patterns in all patients

Line of therapy is denoted by the number followed by the treatment regimen, with first line on the left and subsequent lines to the right.
ET: Endocrine therapy.
